# The Science of Racing against Opponents: Affordance Competition and the Regulation of Exercise Intensity in Head-to-Head Competition

**DOI:** 10.3389/fphys.2017.00118

**Published:** 2017-02-28

**Authors:** Florentina J. Hettinga, Marco J. Konings, Gert-Jan Pepping

**Affiliations:** ^1^Centre for Sports and Exercise Science, School of Biological Sciences, University of EssexColchester, UK; ^2^School of Exercise Science, Australian Catholic UniversityBrisbane, QLD, Australia

**Keywords:** athlete–environment interactions, decision-making, pacing, endurance sport

## Abstract

Athlete–environment interactions are crucial factors in understanding the regulation of exercise intensity in head-to-head competitions. Previously, we have proposed a framework based on the interdependence of perception and action, which allows us to explore athletic behavior in the more complex pacing situations occurring when athletes need to respond to actions of their opponents. In the present perspective we will further explore whether opponents, crucial external factors in competitive sports, could indeed be perceived as social invitations for action. Decisions regarding how to expend energy over the race are based on internal factors such as the physiological/biomechanical capacity of the athlete in relation to external factors such as those presented by opponents. For example: Is the athlete able to overtake competitors, or not? We present several experimental studies that demonstrate that athletes regulate their exercise intensity differently in head-to-head competition compared to time-trial exercises: Relational athlete-environment aspects seem to outweigh benefits of the individual optimal energy distribution. Also, the behavior of the opponents has been shown to influence pacing strategies of competing athletes, again demonstrating the importance of relational athlete–environment aspects in addition to strictly internal factors. An ecological perspective is presented in which opponents are proposed to present social affordances, and decision-making is conceptualized as a resultant of affordance-competition. This approach will provide novel insights in tactical decision-making and pacing behavior in head-to-head competitions. Future research should not only focus on the athlete's internal state, but also try to understand opponents in the context of the social affordances they provide.

## The regulation of exercise intensity throughout the years: a focus on understanding how to pace a time-trial

In endurance sports, athletes are continuously required to make decisions about how and when to invest their limited energy resources over time to achieve the completion of one or multiple exercise tasks (Smits et al., 2014). This goal-directed regulation of the exercise intensity over an exercise bout has been defined as “pacing” (Abbiss and Laursen, 2008) and is widely recognized as an essential determinant for performance (Edwards and Polman, 2013). Research has focused on explaining how athletes pace their races and regulate their exercise intensity. Modeling studies have been conducted as early as in the 80's (Van Ingen Schenau, 1980, 1982; Van Ingen Schenau and De Groot, 1983; Van Ingen Schenau et al., 1983, 1990; Van Ingen Schenau and Cavanagh, 1990) demonstrating that in terms of aerodynamics and power losses, a fast start strategy was optimal for time-trials shorter than 2 min. For the longer distances, an even-paced strategy fluctuating around average velocity was advisable (Abbiss and Laursen, 2008). More recently, modeling studies explored optimal pacing in middle-distance time-trials of about 2 min' duration (Hettinga et al., 2011, 2012) while collecting experimental evidence to support their outcomes in reality. The success of a fast start strategy was confirmed. Nevertheless, the studies provided food for thought regarding how fast a fast start should be, and if there would be differences between sports regarding optimal pacing. A subsequent experimental study demonstrated that indeed, pacing differs between sports (Stoter et al., 2016). When instructed to perform optimally with a fast start strategy, cyclists started explosively with a very fast first quartile of the race. Skaters also started fast, but less explosive and spread their energy over a relatively fast first half of the race, with similar muscle fatigue levels compared to cycling at the finish line.

The above provides an example of how modeling studies supported by experimental studies can lead to new insights into pacing and the regulation of energy expenditure. And in addition, many experimental studies have been conducted, manipulating different pacing strategies since the nineties and onwards (Foster et al., 1993, 2003, 2004, 2005; De Koning et al., 2005, 2011), providing interesting insights into actual pacing outcomes. Based on these, as well as on observational studies exploring athletic behavior in competition, several theoretical frameworks have been proposed to understand the process of pacing. In 1996, the model of teleoanticipation was introduced (Ulmer, 1996) and further expanded upon in the central governor theory (Noakes, 1997, 2012) providing the first larger context on how pacing might actually be regulated. The concept of teleoanticipation suggested that the execution of a task is regulated in an anticipatory way, in which a pre-planned strategy or template (Foster et al., 2009) for how the task should be performed is already formed before the start of the race, based on information of the endpoint; a feedback control system must exist, including a programmer that takes into consideration the finishing point of the race (Ulmer, 1996; Noakes, 1997). Since then, other theoretical frameworks were introduced (Marcora, 2008a,b; Edwards and Polman, 2013). However, it has only been very recently that the importance of decision-making aspects and the external environment was emphasized in the context of pacing (Renfree et al., 2014a; Smits et al., 2014). As a result, most previous models have not addressed athlete–environment interactions, and most experimental and modeling studies focused solely on time-trial exercise: racing against the clock. Yet most competitive sport are characterized by head-to-head competitions, where all contenders start at the same time and the winner of the event is the one who passes the finish line first. In head-to-head middle-distance and endurance competition, athlete–environment interactions are crucial in understanding the regulation of exercise intensity (Smits et al., 2014). Previously, we have proposed a framework based on the interdependence of perception and action, which allows us to incorporate, understand, and explore athletic behavior in more complex tactical pacing situations occurring when athletes need to respond to actions of their opponents (Smits et al., 2014). This ecological framework toward pacing argues that the external world provides the athlete with several social invitations for action (Smits et al., 2014). In the following, we will illustrate this concept by focusing on arguably the most important external factor present in competitive sports: the opponent. We will overview studies that have been conducted on racing against opponents, and discuss how these have contributed to understanding the science behind racing against opponents. Finally, we will further elaborate on our previously proposed ecological framework to understand pacing, tactics, behavioral responses, and decision-making of athletes when racing against opponents.

## Does an opponent make a difference at all?

Indeed, the presence of an opponent has been shown to influence performance (Hulleman et al., 2007; Peveler and Green, 2010; Bath et al., 2012; Corbett et al., 2012; Stone et al., 2012; Lambrick et al., 2013; Tomazini et al., 2015; Williams et al., 2015a,b; Jones et al., 2016; Konings et al., 2016c). In general, improved performance is seen during competitive running and cycling trials compared to individual or non-competitive trials (Corbett et al., 2012; Stone et al., 2012; Tomazini et al., 2015; Williams et al., 2015a,b; Konings et al., 2016c). However, the actual presence and perception of the opponent seems to be crucial. Even the prospect of a monetary incentive ($100,-) did not improve 1500 m cycling performance when the “competitor” (i.e., best previous performance so far) was not visible during the trial (Hulleman et al., 2007).

Interestingly, the presence of a second runner did not improve 5-km running performance when the distance between the athlete and second runner was maintained at ~10 m during the time-trial (Bath et al., 2012). The perception of approaching or getting further behind your opponent might even be a crucial variable (Meerhoff et al., 2014). However, starting 1 min behind (chasing) or in front (being chased) of an opponent did not affect performance (Peveler and Green, 2010). Konings et al. (2016c) recently showed that also the actual behavior of the opponent affected pacing behavior (Konings et al., 2016c). That is, a faster starting opponent evoked a faster start than a slower starting opponent. The presence, as well as the behavior of an opponent affect the decision-making process and pacing behavior of an athlete when competing in a race. Previous research has made several suggestions to explain why athletes perform better when an opponent is present. For example, an increased motivation (McCormick et al., 2015) and a change in attentional focus from internal to external aspects have been mentioned (Williams et al., 2015a). Experimentally, it was demonstrated that when an opponent is present, athletes were able to handle higher levels of peripheral fatigue (Konings et al., 2016b).

Also in the field, it was demonstrated that athlete–environment interactions affected athletic decisions and pacing behavior. Observational studies with large datasets and advanced statistics explored different competitive events involving head-to-head competition such as short track speed skating, rowing, running and cycling (Jones and Whipp, 2002; Paton and Hopkins, 2006; Dwyer et al., 2013; Hanley, 2013, 2014, 2015; Moffatt et al., 2014; Renfree et al., 2014b; Edwards et al., 2016; Konings et al., 2016a; Noorbergen et al., 2016). In all these studies, tactical components, such as favorable positioning, drafting, competing for the optimal line, and minimizing fall risk, seemed to influence decisions and draw athletes away from the energetically favorable strategies as would be performed in time-trial exercise. One could even lose a gold medal despite a higher average velocity due to adverse positioning wide on the bend (Jones and Whipp, 2002), particularly in important events such as the Olympic Games and World Championships (Thiel et al., 2012; Renfree and St Clair Gibson, 2013). Only when an all-out strategy could be adopted from the beginning of the race, athletes adopted a comparable pacing strategy to time-trial sports and modeling studies (Hanon and Gajer, 2009; Noorbergen et al., 2016). Interestingly, sports with a high beneficial effect of drafting behind your opponent (e.g., short-track speed skating, cycling) are characterized by a relatively slow development of the race (Moffatt et al., 2014; Konings et al., 2016a; Noorbergen et al., 2016). A remarkable exception is the relatively fast pace adopted in the elimination discipline in track cycling (Dwyer et al., 2013). This might be explained by the unique character of the discipline in which every two laps the last ranked competitor is eliminated.

In contrast, in competitive sports with a relatively low beneficial effect of drafting such as race walking or middle-distance and marathon running, (sub-)elite runners tend to adopt a pacing strategy in the beginning of the race that they cannot sustain until the end of the race (Hanley et al., 2011; Thiel et al., 2012; Hanley, 2013, 2014; Renfree and St Clair Gibson, 2013; Deaner et al., 2015). In addition, the slowdown was higher for men compared to women during marathons (Deaner et al., 2015). In the Oxford-Cambridge Boat race, in which being in second position has negative effects associated with being in the wake of the leading boat, increasing energy costs, a fast start strategy has been employed by all teams since 1890 (Edwards et al., 2016), even though energetically, an even paced strategy would be favorable (Van Ingen Schenau et al., 1983). The team in the lead after the first quartile of the race won the race in 81% of the cases. It thus seems that the possibility of drafting, and the magnitude of associated energy-saving effects of drafting, is an important determinant for pacing behavior and tactical decision-making. It has been proposed that pacing against opponents can be seen as collective behavior (Renfree et al., 2015) in which indeed, drafting benefits are a crucial determinant for behavior. Finally, in certain situations athletes may even decide to cooperate rather than compete with their opponents (Hanley, 2015).

All these examples based on experimental and observational data have demonstrated that racing against an opponent is different from riding a time-trial: balancing tactical (dis)advantages against the energetically optimal distribution pace is required to perform optimally. This is demonstrating the need for a theoretical framework incorporating athlete–(social pacing) environment interactions.

## Perception-action and athlete-pacing environment mutuality

In the following, a framework (based on ecological psychology and first introduced in Smits et al., 2014) is outlined that allows us to incorporate, understand and explore athletic behavior in the more complex tactical pacing situations occurring when athletes need to respond to actions of their opponents.

Since the mechanization of the worldview in the Seventeenth century, mind-body and man-environment dualism have dominated the scientific disciplines (e.g., Lombardo, 1987; Reed, 1996). The separation of mind and body that ensued has had huge consequences for the way in which scientists have conceptualized cognition and how it relates to pacing and decision-making in the regulation of exercise intensity. Most important, the environment in which sport behavior takes place became conceived of as meaningless, consisting merely of matter in motion (see e.g., Neisser, 1967). This ultimately led scientists to become interested in hypothetical constructs like mental sensations, memory, and information processing (the basics of cognitive psychology). These processes have now become more valuable to researchers than the experience of the concrete and real events and behaviors that actually take place in the environment.

In response, in the late 1970's Gibson formulated what would become the foundations of ecological psychology (Gibson, 1977, 1979; Reed, 1996; Gibson and Pick, 2000). This is a theoretical perspective “…in which the psychological experiences and activities of persons and animals are placed firmly at the center of our field” (Reed, 1996, p. 6). It is an approach that has two mutualities at its core: (i) that of athlete and environment, and (ii) that of perception and action. Both these mutuality's come together in the concept of affordances: the opportunities for action that are presented to us by the environment in which we perform. Affordances are meaningful and real relations between athletes and their environments that are perceived directly. They are defined relative to the action-capabilities of an athlete, and allow for prospective control of actions. What is important for understanding decision-making in pacing is that they are dynamic. All (sport) behavior is regulated relative to affordances (for a full account, see Fajen et al., 2009; Barsingerhorn et al., 2012).

Affordances can be characterized as invitations for action from the environment (Withagen et al., 2012). In the context of sport, the environment of an athlete consists of invitations from objects, places and events, and—important for the current perspective—other people, relevant for that athlete. Affordances become available and dissipate, and allow the athlete to make trade-offs between choosing to persist in a given behavior (e.g., remain on current pace, or not respond to an action of the opponent) or switching to a different one (speed up or slow down, or follow the actions of an opponent or even overtake him/her). In this way, the performance environment surrounds athletes with a multitude of invitations for action. It is up to the athlete to act upon certain affordances, and not on others. This leads to the notions of affordance based decision-making and the affordance-competition hypothesis (Cisek, 2007; Cisek and Kalaska, 2010).

Traditionally, decision-making in natural behavior is interpreted as a sequential process in which the selection of a particular behavior (keeping at current pace, speeding up, slowing down, overtake, etc.) occurs before the behavior is specified, or coded by the brain, and to be executed by the body. The theory of affordances proposes a radical departure from this line of reasoning, and suggests that the selection and the specification of behavior are essentially the same dynamic process (Cisek and Kalaska, 2010; Barsingerhorn et al., 2012). The affordance competition hypothesis describes how the interactions between an individual's needs, action capabilities, and the environment provide for the specification of potential affordances (and related actions). From this viewpoint, decision-making should be understood as emerging from specification of simultaneously available affordances, and the competition between them (Smits et al., 2014). Similarly, in understanding how organisms choose between affordances in situations in which different affordances can be utilized, Reed (Reed, 1993) posited that intentional patterns of organization emerge in situations in which organisms have a real choice of behavior. Following a Darwinian line of reasoning, Reed argued that intentions: “are ‘species’ that emerge out of competition among perceptual and action processes for utilizing affordances” (Reed, 1993, p. 65; see also Withagen et al., 2012). A further extensive overview of research that has examined the affordance-competition-hypothesis in neuropsychological and neurophysiological research can be found in Cisek and Kalaska (2010).

## Opponents as social invitations for action

Head-to-head competition takes place in a complex performance environment. Complex, in this context, refers to the notion that the ongoing modulation of behavior during head-to-head competition is embedded in a complex system that comprises the athlete, opponents, as well as a large number of other interacting components (Araújo et al., 2006; Araújo and Davids, 2009). Following the idea of athlete-environment mutuality, the complex performance environment allows athletes to continuously modify ongoing behavior or pre-planned race strategies, on the basis of—and resulting from the competition between—the affordances that are available to them. This includes opportunities for action that are constrained by factors internal to the athlete and for instance dictated by the athlete's energy systems (fatigue (Konings et al., 2016b) and pain (Mauger, 2014; Astokorki and Mauger, 2017). It also includes opportunities for action presented by the athlete's material and technology (such as distance and speed information available from external devices Boya et al., 2014; Smits et al., 2016). But most importantly for our current argument, there are affordances presented by the social environment, such as opponents in a competition or race (Konings et al., 2016c). We propose that opponents present social affordances; social invitations for behavior. In racing against opponents, three categories of social affordances can be identified (see also Fajen et al., 2009). Firstly, there are affordances *for* opponent(s), that is, what opportunities for action are available for opponent(s)? And even, does the perceiving and acting athlete present an opponent with affordances? For example: if an athlete starts very fast, it is likely that he or she “invites” their opponent to start faster than (s)he would when riding alone (Konings et al., 2016c). Secondly, there are affordances *of* an opponent: What actions does an opponent invite the perceiving and acting athlete to do? Related to drafting opportunities for example, decisions are shaped by the opportunities opponents offer in terms of energy efficiency when staying behind them. Another example is overtaking: the decision to overtake is dependent on the actions and proximity of the opponents, an interesting aspect for further research (Al et al., 2016; de Jong et al., 2016; Hettinga et al., 2016). Finally, there are affordances for *joint action*. These are the opportunities for action available to a group or system as a whole. There are likely to be differences between pacing behaviors in situations where pacing is construed or imposed, for instance through the use of a pace-setter or rabbit in track-and field running, and those instances where pacing behavior is more “organic.” The collective behavior patterns as those described to emerge in peloton formations, such as peloton phase transitions, phase symmetry, peloton divisions, and between-rider distances (Trenchard et al., 2014; Renfree et al., 2015) are related to affordances for joint action.

## Implications, recommendations, and conclusions

To conclude, we have seen how opponents influence performance and make a difference to the pacing strategies that are enacted. A framework is presented that provides an alternative way of understanding decision-making regarding the regulation of exercise intensity in social contexts. An approach, rooted in the affordance-competition hypothesis as described in Smits et al. (2014) is put forward. Perception-action and athlete–environment interactions are a crucial factor in understanding the regulation of exercise intensity in head-to-head middle-distance and endurance competition. Decision-making is the actualization of affordances, and a result of the competition between simultaneously available affordances. Decisions regarding how to expend energy over the race are based on relationships between internal factors such as the physiological/biomechanical capacity of the athlete and external factors such as opponents. For example: Is the athlete able to accelerate fast enough to overtake competitors? How does fatigue impact on the decision to accelerate?

Decision-making, conceptualized as resulting from the competition between affordances, implies that not all affordances presented at a certain moment in time—however effective they may seem—are actualized. In the competition between affordances, only certain survive. For instance, a fast-starting (group of) cyclists might afford other cyclists a similar fast start. With experience and training, an athlete can learn to discover other affordances, for instance that a fast-starting (group of) cyclists requires an alternative response, because of the negative effects it can have on pacing. This resisting of certain affordances in competition might be difficult for the athlete, but it is an important skill to learn with regard to pacing. In a similar vein, not all affordances invite. As argued by Withagen et al. (2012, p. 255): “Although (experienced) affordances can have the potential to invite a certain activity, the vast majority of affordances do not. […] a single object generally affords multiple behaviors to an individual, and not all of these affordances invite.”

When in competition, opponents present a multitude of affordances that influence motivation, attentional focus (perception), the ability to tolerate fatigue and pain, positioning, drafting, falls risk and collective behavior. The presented framework explains that athletes' decision-making cannot and should not be understood in the independent understanding of these aspects. Rather, decision-making, and modification of ongoing behavior can only be properly understood in the context of the simultaneous availability of multiple affordances (related to opponents, fatigue, positioning, etc.) and the competition between them. For future research into the effect of opponents on the regulation of exercise intensity it is therefore advised to understand opponents in the context of the *social affordances* that they provide and the changes they invite in the ongoing behavior of athletes. At the same time, internal aspects are crucial, and we need to understand how and to what extent internal and external factors interact. Studies of competitive behavior in the field are crucial here, but also elegantly designed experiments manipulating both external factors (such as athlete behavior) as well as internal aspects (such as fatigue).

## Author contributions

FH and GP contributed to conception and design of the work, drafted it and revised it critically for important intellectual content. All authors (FH, MK, GP) contributed to the final draft and have approved the final version of the manuscript, agree to be accountable for all aspects of the work in ensuring that questions related to the accuracy or integrity of any part of the work are appropriately investigated and resolved. All persons designated as authors qualify for authorship, and all those who qualify for authorship are listed.

### Conflict of interest statement

The authors declare that the research was conducted in the absence of any commercial or financial relationships that could be construed as a potential conflict of interest.
